# Prevalence rates of health and welfare conditions in broiler chickens change with weather in a temperate climate

**DOI:** 10.1098/rsos.160197

**Published:** 2016-09-07

**Authors:** Chérie E. Part, Phil Edwards, Shakoor Hajat, Lisa M. Collins

**Affiliations:** 1 School of Biological Sciences, Queen's University Belfast, Medical Biology Centre, 97 Lisburn Road, Belfast BT9 7BL, UK; 2 Department of Population Health, London School of Hygiene and Tropical Medicine, Keppel Street, London WC1E 7HT, UK; 3 Department of Social and Environmental Health Research, London School of Hygiene and Tropical Medicine, 15-17 Tavistock Place, London WC1H 9SH, UK; 4 School of Life Sciences, University of Lincoln, Brayford Pool, Lincoln LN6 7TS, UK

**Keywords:** animal health, animal welfare, broiler chicken, epidemiology, climate, temperature

## Abstract

Climate change impact assessment and adaptation research in agriculture has focused primarily on crop production, with less known about the potential impacts on livestock. We investigated how the prevalence of health and welfare conditions in broiler (meat) chickens changes with weather (temperature, rainfall, air frost) in a temperate climate. Cases of 16 conditions were recorded at approved slaughterhouses in Great Britain. National prevalence rates and distribution mapping were based on data from more than 2.4 billion individuals, collected between January 2011 and December 2013. Analysis of temporal distribution and associations with national weather were based on monthly data from more than 6.8 billion individuals, collected between January 2003 and December 2013. Ascites, bruising/fractures, hepatitis and abnormal colour/fever were most common, at annual average rates of 29.95, 28.00, 23.76 and 22.29 per 10 000, respectively. Ascites and abnormal colour/fever demonstrated clear annual cycles, with higher rates in winter than in summer. Ascites prevalence correlated strongly with maximum temperature at 0 and −1 month lags. Abnormal colour/fever correlated strongly with temperature at 0 lag. Maximum temperatures of approximately 8°C and approximately 19°C marked the turning points of curve in a U-shaped relationship with mortality during transportation and lairage. Future climate change research on broilers should focus on preslaughter mortality.

## 1. Background

The Earth's climate is changing. Over the next century, we should expect temperatures to rise, particularly in the mid–high latitudes of the Northern Hemisphere [1]. Europe and North America should also prepare for more extreme rainfall [2–4] and more frequent, intense and longer lasting heat waves [5–7]. Such changes are expected to impact on human mortality, with more heat-related, and less cold-related, deaths predicted over coming decades [8,9].

Non-human animals are also likely to feel the effects, and farm animals might be particularly vulnerable due to genetic selection and captive living conditions. As our climate changes, the livestock industry could face increased mortality and susceptibility to disease, with reduced animal welfare and productivity [10–14]. However, to date, impact assessment and adaptation research in the agricultural sector has focused primarily on crop production, with livestock health and welfare considered mainly in terms of climate change mitigation [12].

Animal welfare is defined as ‘how an animal is coping’ with its living conditions, and ‘refers to the state of the animal’ itself, which is critically related to the health of that animal ([15], p. 1). But, poor health and welfare in livestock has consequences beyond those felt by the animals themselves. It is important from both an ethical and economic standpoint, with further implications for human health and future food security. Some health conditions are linked with bacteria, such as *Escherichia coli*, and may pose a risk to human health if affected carcasses were to enter the food chain [16]. Resulting condemnation of affected carcasses at slaughter/processing detract from farmer profit and add to food waste, both directly and indirectly, through meat and feed (i.e. crop) losses, respectively.

Globally, meat production has increased fourfold within the past five decades.^1^ Much of this increase is due to a rise in poultry consumption, and this trend is expected to continue [17]. Poultry is now the second most popular source of meat worldwide^2^ and, with over 61 billion individuals slaughtered for their meat in 2013 alone,^3^ chickens (*Gallus gallus domesticus*) are by far the most common farm animal on the Earth^4^ [18].

In today's conventional farming systems, broiler (meat) chickens live out their short lives (less than 40 days for most) indoors: confined to the floor of large man-made ‘broiler houses’, specifically designed to control the birds' environment in order to maximize productivity [18,19]. Despite indoor climate control systems, seasonal patterns have been observed in the health and welfare of commercial broiler flocks. In temperate climates, the incidence and severity of footpad dermatitis (FPD)—a skin condition linked to birds' contact with wet litter and characterized by inflammation and necrotic lesions on the footpads and toes [20]—is generally greater during winter than summer months [21–25]. On the other hand, cellulitis—a condition caused by bacteria entering an open skin wound, leading to inflammation of the deep subcutaneous tissues [26]—is generally more common during summer than winter months.^5^

In theory, animals reared on intensive indoor farms should be less vulnerable to meteorological conditions, and to the direct effects of climate change, than grazing animals; however, this is wholly dependent on the capacity and efficiency of internal climate control systems [12,13]. Observed seasonal patterns in the incidence of FPD and cellulitis in commercial broiler flocks tentatively suggest that indoor systems might not provide as much protection from meteorological conditions as initially assumed. Indeed, in northern temperate regions, the welfare of intensively farmed livestock may be at greater risk from projected climate change than that of extensively farmed livestock, at least in terms of imposed thermal challenges [12,13].

Given the far-reaching consequences of poor health and welfare in livestock, along with the current scale (and projected rapid growth) of the chicken meat industry [17], climate change impact assessment and adaptation research on broilers should be prioritized. However, we must first understand the relationships between current weather patterns and broiler health/welfare. Using data collected from more than 6.8 billion individuals over the course of 11 years, this paper addresses the question of how prevalence rates (PRs) of health and welfare conditions, identified in broiler chickens at slaughter/processing, change with weather (temperature, rainfall and air frost) in a temperate climate. We tested the broad hypothesis that changes in weather are associated with changes in the prevalence of conditions, with the aim of directing future climate change impact and adaptation research efforts. Taking Great Britain (GB) as broadly characteristic of the northern temperate climate [13], we set out to: (i) provide current PRs of 16 health and welfare conditions in broiler chickens; (ii) examine the distribution of conditions across time and space; and (iii) describe their associations with recent weather patterns.

## 2. Material and methods

### 2.1. Datasets

#### 2.1.1. Slaughterhouse data

Datasets were shared by the Food Standards Agency (FSA), an independent Government body that is responsible for food safety throughout the UK. Data are collected by FSA to monitor the occurrence of animal health/welfare conditions, and to identify situations in which animal welfare has been compromised. When flock incidence rates exceed set threshold levels, communicative reports are generated for the producer and competent authority (i.e. Animal and Plant Health Agency, UK), and actions are taken to resolve or rectify the issue/s, in line with Council Directive 2007/43/EC [27].

Data collected after transposition of Council Directive 2007/43/EC [27] (i.e. from 1 July 2010 onwards) were available at batch level (see §2.1.1.3), and included the location at which each batch had been reared (i.e. producer/farm postcode). Longer term data (spanning back to January 2003) were only available as monthly totals per slaughterhouse (number slaughtered and counts of each health/welfare condition; see §2.1.1.2), with no corresponding food chain information. Both datasets were shared by FSA, and each was used for different analyses herein. National PRs and distribution mapping were based on batch-level data from more than 2.4 billion individuals, collected between January 2011 and December 2013. Whereas, analysis of temporal distribution and associations with national weather were based on monthly data from more than 6.8 billion individuals, collected between January 2003 and December 2013.

*2.1.1.1. Data collection*. Cases (counts) of each condition (table 1) were identified and recorded during ante- and post-mortem inspections at approved poultry slaughterhouses in GB. The purposes of these inspections are: (i) to ensure food safety and quality; (ii) to detect conditions of significance to public or animal health; and (iii) to identify animal welfare concerns [44].

**Table 1.** Details of monthly slaughterhouse data shared by the FSA, including the working labels under which conditions were recorded and the number of establishments that reported each condition (*n*_e_).

	time span				
condition labels^a^	from	to	definitions based on current condition labels	resulting condemnation	*n*_e_
abnormal smell, colour (bleeding, jaundice)	Jan 2003	Apr 2008	virulent bacterial invasion of the bloodstream [28]	total rejection of carcass and offal [28]	77
*superseded by: **abnormal colour/fevered***	May 2008	Dec 2013			
***ante-mortem rejects (cull/runts)***	Jan 2006	Dec 2013	birds that are significantly smaller than the flock average [29]	culled at hang-on point OR passed as fit for human consumption [29]	48
***ascites/oedema***	Jan 2003	Dec 2013	abnormal accumulation of fluid in the abdomen [30]	total rejection of carcass and offal [30]	75
***cellulitis***	May 2008	Dec 2013	inflammation of the connective tissue between the skin and muscle caused by infection [31]	partial OR total rejection (latter if lesions are not clearly localized or accompanied by systemic effects) [31]	28
dead on arrival	Jan 2003	Jul 2010	broilers that are found dead at hang-on point or in the lairage [32]	total rejection of carcass and offal [32]	75
*re-labelled*: ***dead on arrival/dead in lairage (DOA/DIL)***	Aug 2010	Dec 2013			
emaciation/cachexia	Jan 2003	Apr 2008	birds of all sizes that have very poor muscle development and little or no fat deposits [33]	total rejection of carcase and offal [33]	77
*re-labelled*: ***emaciation***	May 2008	Dec 2013			
***hepatitis***	Jan 2006	Dec 2013	inflammation of the liver, which may be toxic or infectious in origin [34]	partial OR total rejection (latter if other organs and/or carcass are affected) [34]	56
joint lesions/arthritis/ tenosynovitis	Jan 2003	Apr 2008	inflammation of joint/s; shortening and thickening of long bones and lateral slipping of tendon/s; linear twisting of long bones [35]	partial OR total rejection (latter if signs of systemic infection) [35]	61
*superseded by*: ***joint lesions***	May 2008	Dec 2013			
***other farm*** (jaundice, oregon, white muscle, congenital malformations)	Aug 2009	Dec 2013	jaundice: yellow discoloration of the skin, body fat, mucous membranes and internal organs, caused by accumulation of bilirubin. Oregon: green discoloration of deep breast muscle. White muscle: white stripes in breast muscle. Congenital malformations: physical defect caused by a genetic factor [36]	partial (Oregon and congenital malformations) OR total rejection (jaundice and white muscle) [36]	25
***pericarditis***	Jan 2003	Dec 2013	inflammation of the pericardium (sac surrounding the heart) [37]	partial OR total rejection (latter if associated secondary condition or *Salmonella Enteritidis*/*S. Typhimurium*) [37]	67
***perihepatitis/ peritonitis***	Jan 2003	Dec 2013	inflammation of the liver capsule/yellow pus or dry cheese-like exudates limited to the abdominal cavity [38]	total rejection of carcase and offal [38]	74
respiratory disease (air sacculitis, sinusitis, rhinitis)	Jan 2003	Apr 2008	inflammation of the air sacs usually with yellowish caseous exudates in the sacs [39]	partial (if chronic lesions can be removed completely) OR total rejection (if acute lesions or other conditions) [39]	58
*superseded by: **respiratory disease (air sacculitis)***	May 2008	Dec 2013			
***salpingitis***	Jan 2003	Dec 2013	inflammation of the oviduct, which may contain liquid or caseous exudate [40]	partial (if localized lesion) OR total rejection (if secondary condition) [40]	51
skin lesions (ulceration, breast blisters, abscess)	Jan 2003	Apr 2008	inflammation of the skin, often associated with bacterial infection within the skin thickness [41]	partial (if localized lesions) OR total rejection (if generalized condition) [41]	69
*superseded by: **dermatitis***	May 2008	Dec 2013			
trauma (bruising, fractures, dislocations)	Jan 2003	Apr 2008	broken bone/s and/or accumulation of blood [42]	partial (if localized) OR total rejection (if severe and extensive) [42]	73
* superseded by: **bruising/fractures***	May 2008	Dec 2013			
tumour (leukosis, Mareks)	Jan 2003	Apr 2008	abnormal tissue growth in which the multiplication of cells is uncontrolled and progressive. Marek's disease: tumours of the feather follicles, iris, or in more than one viscera [43]	partial (if localized and benign) OR total rejection (in most cases and in the extensive cutaneous and visceral forms of Marek's disease) [43]	60
*superseded by: **tumours/nodules***	May 2008	Dec 2013			

^a^Batch-level data labels in ***bold italics***.

Ante-mortem inspections were carried out by the Official Veterinarian (OV) at each establishment within 24 h of birds' arrival time and less than 24 h before slaughter [44]. Birds were moved from lairage to the slaughter line only after ante-mortem inspections were completed, recorded and signed by the OV.

Post-mortem inspections were typically carried out by Meat Hygiene Inspectors (MHIs) and/or Plant Inspection Assistants (PIAs) working under the supervision of an OV. Each carcass and accompanying offal was inspected without delay after slaughter [44]. Generally, establishments processing less than 3600 birds/hour had one post-evisceration inspection point; those processing 3600–7200 birds/hour had one pre- and one post-evisceration inspection point; and establishments processing more than 7200 birds/hour had one pre- and two post-evisceration inspection points. All inspection posts were positioned for optimum view of carcasses and accompanying offal [45]. Data (counts of each condition; table 1) were recorded by the OVs or MHIs (or PIAs in the case of post-mortem inspections) [44].

*2.1.1.2. Monthly data*. Monthly data spanned 11 years (January 2003–December 2013, inclusive). Eighty-one approved poultry meat establishments in GB (65 in England, nine in Scotland and seven in Wales) contributed to this dataset, reflecting a total of 6 871 486 181 broiler chickens inspected. The total number of birds slaughtered/processed and total number of cases of each condition were provided for individual establishments on a monthly basis. Further details are provided in table 1.

Data were cleaned and formatted prior to analyses. Formatting produced less than or equal to 81 time-series for each condition (i.e. one per reporting establishment). Cases of each condition were then totalled for all reporting establishments on a monthly basis to produce 16 time-series (i.e. one per condition).

*2.1.1.3. Batch-level data*. Batch-level data spanned 3 years (1 January 2011–31 December 2013, inclusive) and included every batch of broiler chickens that was processed in FSA-approved poultry meat establishments in GB during that period. Each batch comprised birds reared on the same farm by the same producer, and processed as a flock at the same establishment. Data were collected in 111 establishments, from 376 776 batches (totalling 2 433 890 777 chickens), reared on 1534 farms (based on producer postcode information) across England, Scotland and Wales.

Counts of 16 health and welfare conditions were provided at batch-level under the labels given (in ***bold italic*** text) in table 1. Accompanying information included: (i) total number slaughtered/processed in batch, (ii) date of slaughter, (iii) batch identification number (ID), (iv) slaughterhouse ID, (v) producer ID; and (vi) producer postcode.

The total number of chickens slaughtered/processed in each batch ranged from 2 to 97 696 (median = 5535 birds). The median age at slaughter was 39 days (interquartile range = 35–45 days). Chickens reared in intensive indoor systems constituted the large majority (more than 96%) of those slaughtered, with considerably fewer broilers reared in extensive indoor (0.07%), free-range (2.71%), organic (0.45%) and ‘other’ systems (0.36%). Most broilers (61.4% of those slaughtered) had been produced at an on-farm stocking density of 33–39 kg m^−2^, with fewer (21.12%) at a lower stocking density (up to 33 kg m^−2^), and considerably fewer (0.55%) at a higher density (39–42 kg m^−2^). The stocking density was unknown for 16.93% of broilers slaughtered.

Data were cleaned and formatted prior to analyses. The following were excluded: (i) batches that were slaughtered in GB but reared elsewhere (number of batches, *n*_b_ = 25; number of chickens, *n*_c_ = 104 863), (ii) batches with no corresponding producer postcode information (*n*_b_ = 5; *n*_c_ = 16 451), (ii) batches where number slaughtered = 1 (likely ‘test batches’ in the system; *n*_b_ = 63; *n*_c_ = 63) and (iv) batches where the number of cases of a single condition > number slaughtered, reflecting error in recording (*n*_b_ = 660; *n*_c_ = 48 649). All producer postcodes were checked against comprehensive datasets of UK postcodes [46,47]. Errors in the recording of producer postcodes were corrected, provided that: (i) corrected postcodes were not listed in either Ordnance Survey [47] or Bell's [46] datasets and (ii) only minor corrections (reflecting obvious typing/input errors) were required to match another producer postcode, with the same unique producer ID, contained within the same dataset.

#### 2.1.2. Weather data

Monthly weather data (© Crown copyright, Met Office) was sourced from publically available archives [48], and spanned 11 years and two months (November 2002–December 2013, inclusive) to reflect the weather experienced on-farm and at slaughter. Four variables (mean daily maximum temperature, mean daily minimum temperature, total rainfall and days of air frost) were downloaded for 29 open weather stations across mainland GB, providing 116 time-series (i.e. one per weather variable per station). Monthly averages for mainland GB were calculated, using available data from all 29 stations, to produce a single (national-level) time-series for each weather variable (*n* = 4).

### 2.2. Data analyses

Unless otherwise specified, data analyses were facilitated by Microsoft Excel^®^ 2010 (Microsoft Corporation) and IBM^®^ SPSS^®^ Statistics, v. 22.

#### 2.2.1. National annual prevalence rates (PRs) of health and welfare conditions

National PRs of each condition in GB-reared broiler chickens at the time of slaughter were calculated from batch-level data, per 10 000 processed, for years 2011, 2012 and 2013: Annual PR = (total number of cases of [condition] identified at slaughter between 1 January and 31 December [year]/total number of broilers processed between 1 January and 31 December [year]) × 10 000.

For presentation purposes (i.e. order of listing in table 2), the weighted average annual prevalence rate of each condition was calculated:

**Table 2.** National annual PRs of health and welfare conditions in GB-reared broiler chickens, per 10 000 slaughtered.

	year		
condition	2011	2012	2013
ascites	26.46	30.25	33.01
bruising/fractures	30.36	24.54	29.14
hepatitis	22.81	25.12	23.32
abnormal colour/fevered	22.10	22.68	22.08
cellulitis	11.35	15.05	18.03
DOA/DIL	12.81	13.19	14.25
perihepatitis/peritonitis	8.06	8.32	9.35
ante-mortem rejects (culls/runts)	2.82	4.52	5.40
pericarditis	4.68	3.97	4.07
emaciation	4.12	3.36	3.13
other farm-related conditions	n.a.	1.46	3.79
joint lesions	2.16	2.77	2.92
tumours/nodules	1.94	1.97	2.14
dermatitis	2.39	1.91	1.68
respiratory disease	1.70	0.32	0.27
salpingitis	0.04	0.07	0.03
no. slaughtered	794 151 084	813 413 852	826 325 841

Conditions listed in descending order of weighted average (2011–2013) annual rates. Based on FSA data.

Weighted average annual PR = ((2011 annual PR of [condition] × total number of broilers processed in 2011) + (2012 annual PR of [condition] × total number of broilers processed in 2012) + (2013 annual PR of [condition] × total number of broilers processed in 2013))/total number of broiler processed between 1 January 2011 and 31 December 2013.

#### 2.2.2. Spatial distribution of conditions

**Figure 1.**
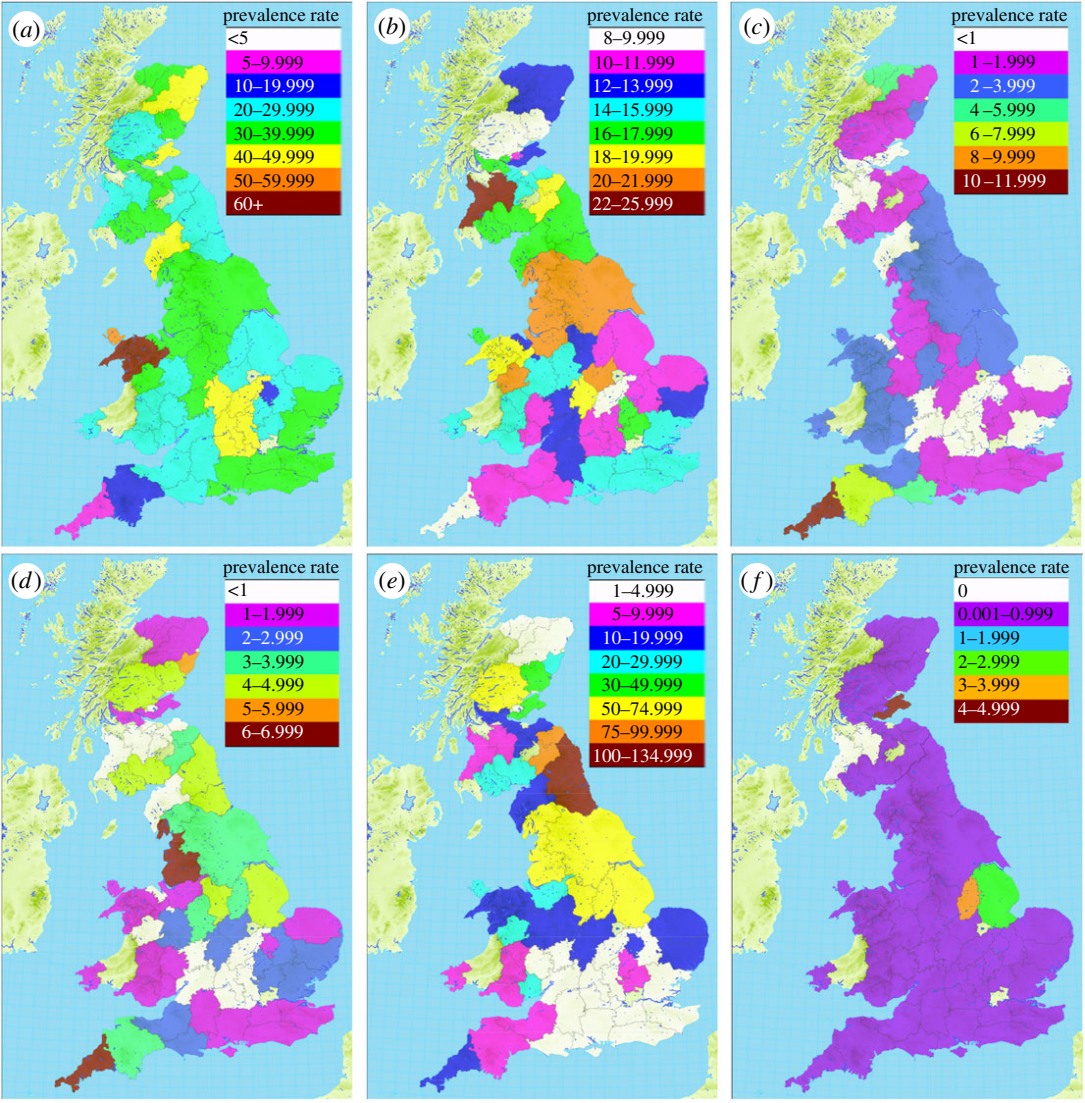
*County-level* PRs of (*a*) ascites, (*b*) DOA/DIL, (*c*) dermatitis, (*d*) joint lesions, (*e*) bruising/fractures and (*f*) respiratory disease in GB-reared broilers, per 10 000 slaughtered, 2011–2013. Data source: FSA. Contains Ordnance Survey data © Crown copyright and database right 2014. Contains data provided by the Historic County Borders Project, available from http://www.county-borders.co.uk. Contains data provided through www.VisionofBritain.org.uk and used historical material which is copyright of the Great Britain Historical GIS Project and the University of Portsmouth. Additional sources: [51,52]. This work made use of Royal Mail data © Royal Mail copyright and database right 2014. This work made use of National Statistics data © Crown copyright and database right 2014. This work made use of data compiled by C Bell [46], available at www.doogal.co.uk (Note: *County-level* is in order to maintain producers' confidentiality, PRs are presented for the following county-combinations: Ayrshire and Lanarkshire; Kirkcudbrightshire and Dumfries shire; East Lothian, Midlothian, West Lothian and Peebles shire; Clackmannanshire and Stirlingshire; Roxburghshire and Berwickshire; Northumberland and Durham; Kent, Surrey, Sussex and Hampshire; Bedfordshire and Hertfordshire; Berkshire and Oxfordshire; Caernarfonshire, Merionethshire and Denbighshire; Brecknockshire, Glamorgan, Radnorshire and Pembrokeshire; Nairnshire, Moray and Banffshire; Westmorland and Yorkshire. In all other cases, PRs are presented at singular county level).

Farms (i.e. producer postcodes) were located within county boundaries [49,50] using ArcMap™ on ArcGIS^®^ for Desktop, version 10.1 (Esri^®^). In order to preserve producers' confidentiality, where less than 5 producer postcodes and producer IDs were located in any one county, this county was combined with the closest county, in the same country, where additional postcodes (less than 5 where possible) were located. Where the combination of two counties did not result in greater than or equal to 5 postcodes and producer IDs, the next closest county was also combined. In some cases, two or three counties (with a small number of postcodes located in each) were combined with a central county where greater than or equal to 5 postcodes were located (see ‘Note’ in figure 1 caption for county-combinations).

Batch-level data were then reduced to county-level, and PRs calculated per 10 000 processed: County/county-combination PR = (total number of cases of [condition] identified in batches from postcodes located in [county/county-combination] between 1 January 2011 and 31 December 2013/total number of broilers slaughtered from postcodes located in [county/county-combination] between 1 January 2011 and 31 December 2013) × 10 000.

Postcodes for which geographical mapping information could not be found (i.e. postcodes not listed in Ordnance Survey [47] or Bell's [46] datasets, *n* = 43) were excluded from county-level calculations. Thus, distribution maps were based on data collected in 110 establishments, from 371 891 batches (totalling 2 399 810 730 chickens), reared on 1491 farms.

#### 2.2.3. Temporal distribution of conditions

Temporal analyses were based on monthly slaughterhouse data, per 10 000 processed: Monthly PR = (total number of cases of [condition] identified at slaughter during [month, year]/total number of broilers processed in 81 reporting establishments during [month, year]) × 10 000.

Time-series graphs and correlograms were visually examined for evidence of long-term and cyclical patterns in each PR series. Linear and curvilinear (higher order polynomial, exponential and power) trends were estimated for each series that demonstrated long-term changes (where dependent variable = PR; independent variable = time). The best model was determined by comparing: (i) *R*^2^ values, (ii) time-series plots with superimposed trends, and (iii) time-series plots of trend fit errors. PR series were then detrended by calculating residuals of the best-fitting trend. Detrending was carried out on each PR time-series that showed evidence of long-term trends (12 of 16 PR time-series, excluding DOA/DIL, respiratory disease, salpingitis and skin lesions/dermatitis) in order to highlight any cyclical or seasonal patterns in these series. Had time-series with evident long-term trends not been detrended, the long-term trend component would be the dominant feature seen in the correlograms, making it more difficult to identify any cyclical or seasonal patterns [53].

Residual series were examined for normal distribution, homoscedasticity across time and independence of data using the Shapiro-Wilk test, Levene test for homogeneity of variance (with year as levels of the independent variable), and Ljung-Box Q tests alongside visual examination of correlograms with 95% confidence intervals, respectively. Where residual series did not fulfil the assumptions of regression analysis, the *F* ratio and corresponding *p*-value of the trend fit were not reported.

Where an additional pattern to the removed trend was indicated by significant Ljung-Box Q statistics, lagged autocorrelations among residuals were examined for evidence of annual cycles. The above procedures were based on the preliminary methods of Warner [53].

#### 2.2.4. Associations between weather and health and welfare in broiler chickens

Time-series graphs and scatterplots of monthly weather (averaged for mainland GB) and monthly PRs (detrended series for scatterplots) were visually examined for signs of linear and nonlinear association. The use of national-level data was required because monthly slaughterhouse data could not be reduced to regional-level, but provided a means of analysing PR patterns over a longer time-frame than that covered by batch-level data.

Relationships between each possible weather-condition combination were tested by Spearman's rank order correlation (*r*_s_) (or Pearson product-moment correlation, *r*, in the small number of cases where the assumptions of this test were met). Where applicable, only detrended PR series were analysed in order to prevent artificial inflation of the correlation coefficient, which can occur if both series show similar long-term trends. To tentatively explore potential timing effects of weather, associations between each weather-condition combination were tested with weather at lags of 0, −1 and −2 months.

Using the total number of broilers slaughtered per month (i.e. monthly throughput) as a crude proxy for on-farm stocking density (i.e. number of birds per m^2^ of floor space), the relationship between monthly throughput and monthly PRs of ascites (detrended series) was also examined by the methods outlined above.

Given the large number of statistical tests, and the very strong correlation between the mean daily maximum temperature and mean daily minimum temperature series (*r*_s_ = 0.97, *p* < 0.001), correlations between PRs and minimum temperature were not tested. Bonferroni correction was also applied to reduce the likelihood of type I errors, where the *p*-value of *r*_s_ was set at: 0.05/145 ((16 conditions × 3 weather variables × 3 lags) + ascites PR/total slaughtered correlation) = 0.0003.

## 3. Results

Owing to the large number of analyses that were carried out; henceforth, only the most noteworthy findings (unless otherwise stated) are presented/discussed in detail.

### 3.1. Annual prevalence rates (PRs) of health and welfare conditions

Annual PRs of health and welfare conditions identified in GB-reared broilers at slaughter/processing are shown in table 2. The most prevalent conditions between 2011 and 2013 were ascites, bruising/fractures, hepatitis and abnormal colour/fever, at (weighted) average rates of 29.95, 28.00, 23.76 and 22.29 per 10 000 processed, respectively. On average, each of these four conditions affected between 1.8 and 2.4 million broiler chickens per year. Tumours/nodules, dermatitis, respiratory disease and salpingitis were the least prevalent conditions, at (weighted) average rates of 2.02, 1.99, 0.75 and 0.05 per 10 000, respectively.

### 3.2 Spatial distribution of conditions

Here, we focus on six conditions believed to be those most likely influenced by the internal or external environment (i.e. ascites, DOA/DIL, dermatitis, joint lesions, bruising/fractures and respiratory disease). See File 1 (Sheet b) of the electronic supplementary material for county-level PRs of other conditions not mentioned below.

The prevalence of ascites was generally consistent across space, at county-level rates of 20–49.99 cases per 10 000 processed (figure 1*a*). However, a relatively higher rate was found in broilers from farms in North Wales (particularly Caernarfonshire–Merionethshire–Denbighshire, at 60.97 per 10 000), and a lower rate in broilers from farms in southwest England (specifically Cornwall, at 8.67 per 10 000). Farms in Cornwall also produced proportionately less broilers that were found DOA/DIL (9.33 per 10 000; figure 1*b*), and proportionately more broilers with dermatitis (11.01 per 10 000; figure 1*c*) and joint lesions (6.57 per 10 000; figure 1*d*), than farms in any other GB county. However, the range of county-level PRs was considerably lower for DOA/DIL (9.33–25.22 per 10 000), dermatitis (0.3–11.01 per 10 000) and joint lesions (0.23–6.57 per 10 000) than for ascites (8.67–60.97 per 10 000).

Bruising/fractures was found to have the largest range in county-level prevalence, from less than 5 per 10 000 in clusters of counties in East Scotland and in Mid-South England to 133.67 per 10 000 in the Northern English counties of Durham–Northumberland (figure 1*e*). Whereas, PRs of respiratory disease were consistently low across GB counties (figure 1*f*). The highest rates were found in broilers from farms in Fife (4.73 per 10 000) in Scotland and in Nottinghamshire (3.43 per 10 000) and Lincolnshire (2.51 per 10 000) in East England, while all other counties had comparable rates of less than 1 per 10 000 processed.

### 3.3. Temporal distribution of conditions

Prior to detrending, the time-series of abnormal colour/fever and trauma (bruising/fractures) were shortened from January 2003–December 2013 (original series) to January 2006–December 2013 and September 2009–December 2013, respectively. This was due to large increases in the prevalence of abnormal colour/fever and trauma injuries observed during January 2006 and August/September 2009, respectively, with subsequent changes in long-term trends. Visual examination of time-series plots displaying monthly PRs and monthly weather factors (not shown) did not reveal any corresponding change in weather at the time of aforementioned increases in abnormal colour/fever or trauma injuries (i.e. January 2006 and August/September 2009, respectively). A push by the FSA to standardize the recording of these two conditions across GB slaughterhouses most probably accounts for the observed increases. Therefore, it was considered best to exclude earlier (pre-standardized) data from the analyses.

**Figure 2.**
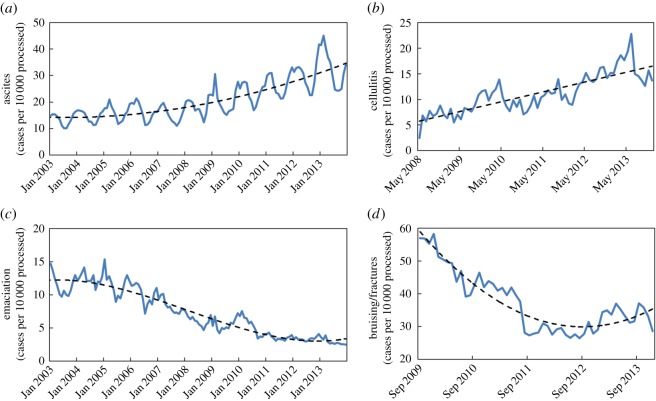
Time-series plots showing monthly PRs of (*a*) ascites, (*b*) cellulitis, (*c*) emaciation and (*d*) bruising/fractures (solid blue lines) with fitted polynomial trends (dashed black lines). Based on FSA data.

As seen from figure 2*a*, the prevalence of ascites showed evidence of exponential growth. Cellulitis also increased in prevalence, approximately linearly (figure 2*b*); whereas, rates of emaciation (figure 2*c*) and bruising/fractures (figure 2*d*) showed exponential decay through time, with some increase in bruising/fracture cases within the last 12–16 months. A quadratic polynomial (*X_t_* = 14.236 − 0.018*t* + 0.001*t*^2^) and linear trend (*X_t_* = 5.637 + 0.16*t*) accounted for 67.7% and 68.6% of the variance in monthly PRs of ascites and cellulitis, respectively, and were removed from the series. A cubic curve (*X_t_* = 12.217 + 0.018*t *− 0.002*t*^2^ + 1.197 × 10^−5^*t*^3^), accounting for 89.8% of the variance, was removed from the emaciation series, and a quadratic curve (*X_t_* = 62.308–1.719*t* + 0.023*t^2^*), accounting for 85.9% of the variance, from the bruising/fracture series. Other conditions did not show such prominent increases or decreases in prevalence (as based on the difference between observed PRs during the first and last months of each analysed series).

**Figure 3.**
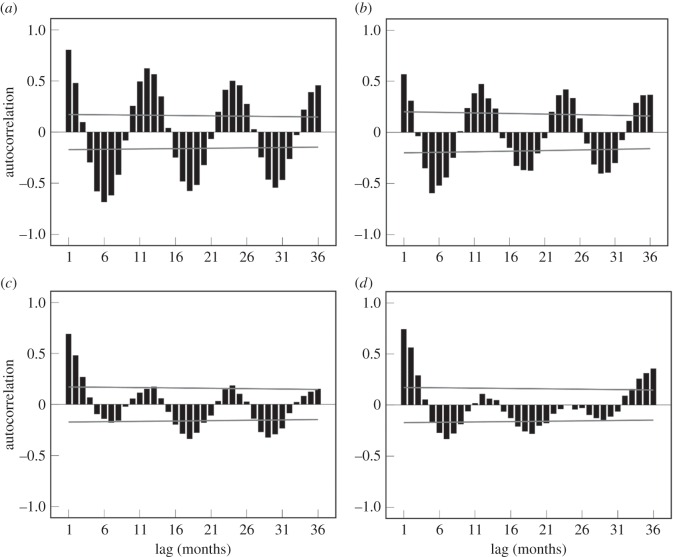
Correlograms showing autocorrelations among (long-term) trend residuals in the monthly series of: (*a*) ascites, (*b*) abnormal colour/fever, (*c*) emaciation and (*d*) perihepatitis/peritonitis. Black bars represent coefficients (i.e. sign and strength of autocorrelation between the first data point in the series and each point thereafter). Grey lines represent 95% confidence intervals. Based on FSA data.

The lagged autocorrelation function of trend residuals, shown in figure 3*a,b*, exhibited clear oscillation, with coefficients peaking at regular 12-month intervals; indicating the presence of 12-month cycles in the prevalence of ascites and abnormal colour/fever, respectively. Lagged autocorrelations among trend residuals in the emaciation (figure 3*c*) and perihepatitis/peritonitis series (figure 3*d*) followed similar, but less pronounced, patterns.

**Figure 4.**
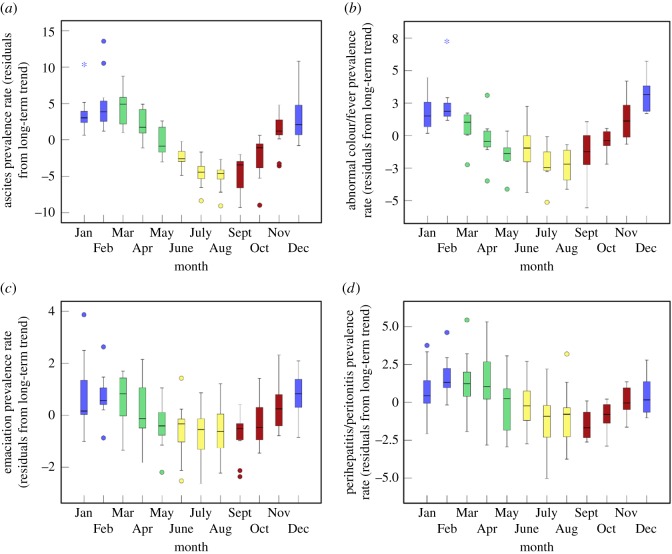
Boxplots summarizing 12-month cycles in the prevalence of (*a*) ascites, (*b*) abnormal colour/fever, (*c*) emaciation and (*d*) perihepatitis/peritonitis at a national level. Plots were based on the residual (i.e. detrended) monthly series. Meteorological winter, spring, summer and autumn seasons are represented in blue, green, yellow and red, respectively. Based on FSA data.

The 12-month cycles are summarized in figure 4, the most notable of which was observed in the ascites series (figure 4*a*), where PRs were consistently higher in winter and early-mid spring months (December–April) than in mid-late summer and early autumn months (July–September). Rates of abnormal colour/fever were consistently higher in winter months than in the summer months of July and August (figure 4*b*). The number of emaciated birds identified at slaughter (figure 4*c*) generally dipped in mid-late spring, rising again in late autumn. Overall, the prevalence of perihepatitis/peritonitis tended to reach a low point in September (figure 4*d*).

There were no clear, significant 12-month cycles in the prevalence of ante-mortem rejects, cellulitis, DOA/DIL, hepatitis, joint lesions, ‘other farm’ conditions, salpingitis, skin lesions/dermatitis or trauma (bruising/fractures). However, the PR of trauma (bruising/fractures) did tend to be lower in August than in other months of the year. There was some evidence of 12-month cycles in the prevalence of pericarditis, respiratory disease and tumours/nodules, but these were somewhat less pronounced than those presented in figure 4.

### 3.4. Associations between weather and health and welfare in broiler chickens

Strong negative correlations were found between average maximum temperature and the prevalence of ascites (*r*_s_ = −0.80*, p* < 0.0003) and abnormal colour/fever (*r*_s_ = −0.75, *p* < 0.0003) in broilers (table 3). The strength of relationship between maximum temperature and abnormal colour/fever decreased with increasing lags (−1 month: *r*_s_ = −0.61, *p* < 0.0003; −2 months: *r*_s_ = −0.29, *p* = 0.004), while the relationship with ascites remained strong at a lag of −1 month (*r*_s_ = −0.84, *p* < 0.0003) and decreased at a lag of −2 months (*r*_s_ = −0.66, *p* < 0.0003).

**Table 3.** Correlation coefficients between detrended monthly PRs of health and welfare conditions at *slaughter* and monthly weather data (mean daily maximum temperature, total rainfall, and days of air frost) for mainland GB, at lags of 0, −1 and −2 months. Coefficients in bold were significant at the 0.0003 level (0.05 level after Bonferroni correction). Dark grey shading represents a strong correlation (*r*_s_ = 0.7–0.9), medium grey represents a moderate correlation (*r*_s_ = 0.5–0.7), and light grey represents a weak correlation (*r*_s_ = 0.3–0.5) [54]. Based on FSA and Met Office [48] data. (Note: *Slaughter*—several series (i.e. DOA/DIL, respiratory disease, salpingitis, skin lesions/dermatitis) were not detrended. Here, correlation coefficients were based on the original monthly prevalence rate series, per 10 000 slaughtered.)

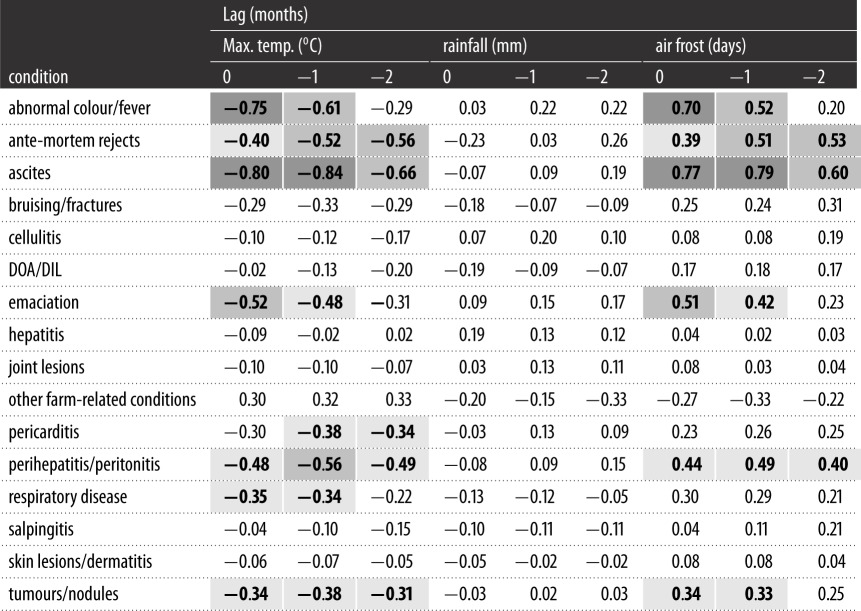

The moderate negative correlation between emaciation and temperature (*r*_s_ = −0.52, *p* < 0.0003) decreased marginally with temperature at a lag of −1 month (*r*_s_ = −0.48, *p* < 0.0003) and more so at a lag of −2 months (*r*_s_ = −0.31, *p* = 0.0004), while the association between perihepatitis/peritonitis and temperature (*r*_s_ = −0.48, *p* < 0.0003) was generally consistent across lags (−1 month: *r*_s_ = −0.56, *p* < 0.0003; −2 months: *r*_s_ = −0.49, *p* < 0.0003). Moderate relationships were also found between the number of ante-mortem rejects at slaughter and maximum temperature at a lag of −1 (*r*_s_ = −0.52, *p* < 0.0003) and −2 months (*r*_s_ = −0.56, *p* < 0.0003), which were stronger than the relationship at zero lag (*r*_s_ = −0.40, *p* < 0.0003).

Positive correlations between days of air frost and prevalence of abnormal colour/fever, ascites, emaciation, perihepatitis/peritonitis and ante-mortem rejects largely mirrored the negative correlations between maximum temperature and these conditions, both in strength and lag-pattern (table 3). No notable relationships were identified between total rainfall and monthly PRs of any health and welfare condition considered herein.

**Figure 5.**
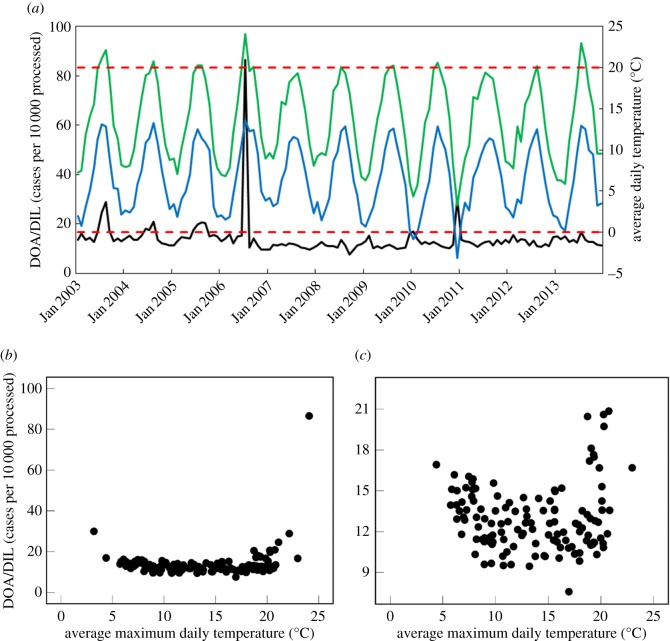
Association between temperature in mainland GB and total number of broilers found DOA/DIL at all reporting GB establishments (*n* = 75), per 10 000 processed: (*a*) time-series plot showing monthly PRs of DOA/DIL (black line), mean daily maximum temperature per month (green line) and mean daily minimum temperature per month (blue line). Red horizontal lines were plotted at 0°C and 20°C; (*b*) scatterplot of number found DOA/DIL, per 10 000 processed and mean daily maximum temperature per month; (*c*) scatterplot of number found DOA/DIL, per 10 000 processed and mean daily maximum temperature per month after removal of major outliers (*n* = 4). Data sources: FSA and Met Office [48]. Data range: January 2003–December 2013.

Correlation analyses indicated no significant linear relationships between weather factors and number of broilers found DOA/DIL at slaughter (table 3). However, the large peak in DOA/DIL rates during July 2006, and smaller peak during August 2003, coincided with rises in maximum temperature above 20°C (24.1°C and 22.1°C, respectively). The peak during December 2010 coincided with the lowest minimum temperature in the series (−3.2°C; figure 5*a*). Indeed, figure 5*b* potentially shows the beginnings of a U-shaped relationship between maximum temperature and number found DOA/DIL; whereby, rates remained stable with temperatures between approximately 8°C and approximately 19°C, which marked the points of curve for exponential increases in DOA/DIL rates. Here, the relationship was being driven by the extreme peaks in broiler mortality during the heatwaves of 2003 and 2006, and during the cold winter of 2010. When these outliers were removed, however, the U-shape remained (figure 5*c*); whereby, mortality rates began to rise (on average) when maximum temperature exceeded approximately 19°C or fell below approximately 8°C.

No relationship was found between total number of broilers slaughtered per month and monthly ascites PR (*r*_s_ = −0.13, *p* > 0.05).

## 4. Discussion

Our findings supported the hypothesis that changes in weather (specifically, maximum temperature and days of air frost) are associated with changes in the prevalence of several health and welfare conditions identified in broiler chickens at slaughter/processing. In particular, ascites, which has been the cause of an increasing number of carcass condemnations in GB slaughterhouses since 2003 (reaching an annual high of 2.7 million in 2013), was considerably more prevalent in broilers that were produced during the coldest times of the year (winter and early spring) than those produced during the warmest (summer and early autumn). Similar seasonal patterns have been observed in national ascites-related condemnation rates for Canada^5^ [55].

Exposure to cold temperatures has long been known to increase the incidence of ascites in commercial broilers (e.g. [56,57]) by increasing the demand for oxygen in an already demanding system (reviewed in [58–60]). This supports the hypothesis that meteorological conditions are driving the seasonal patterns in ascites PRs. If this hypothesis is true, we would be led to one of two conclusions given that the large majority of GB broilers were reared on intensive indoor farms: either the internal climate control systems in today's broiler houses do not have the capacity to cope with current weather patterns, or the systems are not being used to their full capacity in an attempt to, for instance, optimize productivity (e.g. [61,62]) and/or reduce the costs of production (i.e. heating in the winter).

Such conclusions were strengthened by agreement between previous research on the timing effects of *indoor* temperature, and current findings of strong associations between ascites PR and *outdoor* temperature at both 0 and −1 month lags (representing mid–late life and early–mid life in fast-growing broilers, respectively). Specifically, Groves' [63] research suggested that prolonged exposure to cold temperatures during the first two weeks of life increases broilers' susceptibility to ascites, and further exposure during week four (or later) may advance the clinical manifestations of this condition in predisposed birds. Moreover, Sato *et al*. [64] showed that clinical and pathologic signs of ascites can disappear in affected birds when the indoor temperature is maintained at 20 ± 5°C during the last weeks of life.

Given the prolonged suffering that ascites causes in broilers [59], the large number of individuals who are affected each year, and the considerable amount of feed that is wasted in raising them to slaughter age, it is unacceptable if many ascites cases can be prevented (e.g. [63,65]) or reversed [64] by providing broilers with an appropriate thermal environment while under our care.

Although it is widely accepted that prolonged cold exposure is a major trigger of ascites in broilers, we must not discount other (non-climatic) factors that may have confounded the associations reported herein. In theory, increased consumer demand for chicken meat at particular times of the year might translate into higher on-farm stocking densities, which suppresses growth rate and, consequently, the incidence of ascites [66]. However, we did not find a relationship between ascites PR and number of broilers slaughtered per month. A rise in indoor humidity, brought about by reducing the ventilation in broiler houses in an attempt to prevent adverse cold effects and limit heating costs, most probably explains the higher incidence of FPD in winter [20]. To the best of our knowledge, there is no evidence that house humidity affects the incidence of ascites. However, although cold exposure is considered to be a more significant trigger of ascites than inadequate ventilation [66], several reports have attributed outbreaks of ascites to poor air quality, poor ventilation and to the presence of pathogens (reviewed by Wideman *et al*. [67]); all of which could result from farmers having reduced the ventilation in broiler houses to prevent adverse cold effects and limit heating costs in the winter. On the other hand, broilers reared in cold conditions tend to consume more feed [68], which intensifies the birds' oxygen requirements and can lead to ascites [66]. Thus, it seems very likely that weather is driving the seasonal variation in ascites prevalence, either directly or indirectly (or both).

Like ascites, the prevalence of abnormal colour/fever (septicaemia/toxaemia, also known as sepsis or ‘blood poisoning’) tended to decrease as maximum temperature rose and days of air frost fell. Lagged associations indicated that temperature towards the end of life was more strongly associated with the prevalence of abnormal colour/fever at slaughter than temperature in early life. Higher incidence rates of sepsis and associated fatality have also been observed in humans during the coldest times of the year [69], although potential timing effects are unclear.

The Canadian Food Inspection Agency [70] distinguishes between septicaemia/toxaemia and cyanosis; however, the visual characteristics of both conditions appear to be very similar and, therefore, may be difficult to differentiate on a fast-moving slaughter line. As noted by Boulianne & King [71], a carcass with dark-coloured muscle (and no other lesions) may be condemned under septicaemia/toxaemia in the USA and under cyanosis in Canada. Thus, it is possible that (at least some) cases of abnormal colour/fever recorded in GB slaughterhouses reflect cyanosis, rather than septicaemia/toxaemia, which could explain the timing effects identified herein. Specifically, cold outdoor temperature towards the end of life (during transportation to slaughter) has been identified as a risk factor for cyanosis in turkeys [72]. Chicken carcasses that were condemned for cyanosis in Canadian abattoirs were found to have significantly redder breast meat than controls [71,73], and showed traits of dark, firm and dry meat [73]; all of which have been found in broilers exposed to cold temperatures (less than or equal to 0°C) during transportation to slaughter [74] and all of which (apart from ‘firm’ meat) match the criteria for rejecting carcasses due to septicaemia/toxaemia at post-mortem inspection in GB [28].

Contrary to our findings, seasonal differences in preslaughter mortality (DOA/DIL) have been observed in several countries. In Canada, the highest rates are generally recorded during winter.^5^ In Italy [75] and north Iran [76], the highest rates were recorded during summer. In the Czech Republic, rates peaked in both summer and winter [77], while in the subtropical climate of Brazil, rates were higher in summer and spring than in winter and autumn [78]. Overall, higher rates of preslaughter mortality have been found in The Netherlands (46 per 10 000) [79], Italy (35 per 10 000) [75], Brazil (33 per 10 000) [78], Canada (27 per 10 000)^6^ [80] and the Czech Republic (24.7 per 10 000) [77], as compared to GB (average annual 2011–2013 rate: 13.4 per 10 000).

Weather patterns probably contribute to within-country seasonal differences in preslaughter mortality [75,76,78], but may also explain some between-country variation. Hot and humid conditions in summer are thought to increase the risk of heat-stress during transportation and lairage [75]. Transport under cold [74] and wet conditions [81], on the other hand, may lead to cold-stress, and reducing the ventilation to protect against this can create a ‘paradoxical heat-stress’ on-board [82]. Thus, the harsher winters in Canada, warmer summers in Italy and Iran, and hotter and more humid spring/summers in Brazil, may contribute to the higher incidence of DOA/DIL in these nations when compared with GB. However, international differences in other factors, such as transport distance/duration [77,79] and abbatoir size [75], are also likely to be involved.

Although consistent seasonal patterns were not observed herein, the highest DOA/DIL rates coincided with both high and low temperature extremes, which is consistent with previous findings in The Netherlands [79]. Warriss *et al*. [83] reported that DOA rates increased exponentially as maximum daily temperatures rose approximately above 17°C, while temperatures below this threshold appeared to have little effect. We observed a similar trend of increasing mortality when temperatures reached around 19°C. The apparent 2°C increase in the upper threshold of broilers' thermoneutral zone might be accounted for by the different study populations or may reflect recent improvements in transportation, aimed at combating heat-stress during warm weather. Such improvements would also explain the minimization of summer peaks in DOA/DIL rates that were observed pre-2007 ([83,84]; also see figure 5*a*). That Warriss *et al*. [83] did not detect an increase in preslaughter mortality during cold weather probably reflects the severity of recent winters (2009/2010 and, particularly, December 2010 [85]), which were not included in earlier studies. Indeed, the heavy snowfall during December 2010 [85] may have exacerbated the effects of low temperatures by lengthening journey time between farm and slaughter [79].

Of course, weather cannot explain all variation in condition PRs, as alluded to above. Different systems of production may prevail in certain regions and may contribute to within-country spatial differences in PRs. For example, free-range production predominated in Cornwall, where relatively low rates of ascites and DOA/DIL, and high rates of dermatitis and joint lesions, were evident. Growth rate is directly linked to pulmonary hypertension and to the incidence of ascites in broilers [59,60]. Therefore, the use of slower growing strains on Cornwall farms might have contributed to the lower rates of DOA/DIL and ascites found here. On the other hand, fast-growing strains and/or inadequate pasture management can lead to higher rates of skin conditions and skeletal damage in free-range systems [86], which might have contributed to the relatively higher rates of dermatitis and joint lesions in Cornwall-reared broilers. Whereas, the large range in bruising/fracture cases, with spatial clustering of comparable PRs, might be explained by discrepancies in the skill of catching teams and/or slaughterhouse staff who work in different regions, in the slaughter line environment of different establishments, and/or in the maintenance of catching and/or transportation equipment used by different regional teams [42].

Clearly then, weather is not the only factor associated with condemnation rates. Even ascites PRs, which showed clear seasonal patterns and strong associations with weather, also demonstrated a long-term trend that could not be explained by weather. Detrending the monthly PR time-series was a purposeful attempt to isolate seasonal patterns from the potential effects of other variables, such as genetic selection or gradual improvements in detecting conditions on-farm or during ante- and/or post-mortem inspections. Work is currently underway to describe how factors (such as production system, on-farm stocking density, growth rate and age at slaughter) might influence, and interact with weather to influence, broiler health/welfare at slaughter. The inclusion of such factors was beyond the scope of this paper, but estimations of their potential impacts will further assist the development of climate change adaptation strategies for the livestock industry.

Given the potential food safety and/or quality issues that may arise if conditions are missed at slaughter/processing, the trigger system in place that launches an investigation if flock PRs exceed a set threshold level, and post-mortem inspection verification checks, which are carried out by OVs on a sample of carcasses each day, we can be fairly confident in the reliability of the batch-level FSA data analysed herein. Further, poultry condition cards have been specifically developed by FSA personnel (e.g. Veterinary Managers, Lead Veterinarians, OVs and MHIs), and external experts in the field of poultry medicine, to standardize identification and recording of post-mortem conditions in UK slaughterhouses. Photographs and clear descriptions of defective carcasses, with outcome (i.e. rejection) decisions, provide guidance to post-mortem inspection teams. Nonetheless, assuming equal sensitivity and specificity of the inspection process across all establishments at all times may be a stretch too far, and we should not presume perfect consistency in the recording of all conditions across the board. Therefore, future research that makes use of the batch-level FSA dataset will account for variability between slaughterhouses, as well as variability at the farm-level. In terms of validity, the methods used to identify septicaemia/toxaemia at post-mortem inspection may warrant investigation to ensure that data on ‘abnormal colour/fever’ do not include cases of cyanosis with no systemic infection.

With regard to the monthly dataset analysed herein, it should be noted that all contributing slaughterhouses did not report cases of all conditions (as can be seen in table 1). For example, a relatively small number of establishments reported cellulitis when compared with the number of establishments that reported other conditions, such as ascites or DOA/DIL. Closer inspection of the dataset revealed that many of the smaller slaughterhouses did not report cases of cellulitis, which could signify inadequate reporting of this condition by small establishments, or could be a true reflection of many small slaughterhouses not having received batches from farms that experienced problems with cellulitis. Unfortunately, the level of detail provided in the monthly dataset did not enable us to determine the cause of such differences in reporting between establishments. However, the number of slaughterhouses that contributed to the PR time-series of each condition is not likely to have affected our results. That is, if the PR of a condition follows seasonal patterns and/or is associated with weather, this should be evident regardless of whether the time-series is based on the entire population of GB-reared broilers, or part thereof.

The annual PRs in table 2 were calculated from nationwide data collected from the entire population of GB-reared broiler chickens that reached slaughter each year. Nonetheless, it must be noted that PRs recorded at processing stage may be conservative estimates given that birds manifesting health and welfare conditions on-farm may (and should) be culled immediately in order to end pain and suffering in affected animals, limit economic losses and (where applicable) prevent potential infection to other members of the flock.

Using monthly, national-level, data enabled a broad characterization of the relationships between condition PRs and weather; but, the use of such data may have masked, for example, a stronger curvilinear relationship between maximum daily temperature and DOA/DIL rates where extreme weather events were short-lived and traversed two months. Future efforts should focus on modelling associations between daily PRs at slaughter (particularly ascites, abnormal colour/fever, DOA/DIL) and daily fluctuations in temperature, at a more local level. This will facilitate: (i) forecasting of condemnation rates under alternative climate change scenarios and (ii) the development of strategies to minimize the adverse effects of current weather cycles and future climate change on broiler health/welfare.

### 4.1. Implications

The UK is the largest producer of broiler meat in EU-27,^7^ and has above average rates of condemnation [45], but our findings are not only significant to the UK. The broiler industry is growing across the globe, and poultry is now the leading source of meat in Australia, Brazil, Canada, Russia, South Africa, the UK and the USA.^2^ With future food security under threat by climate change and an ever-increasing human population [87], we need to reduce food waste as much as possible [88], including farm animal mortalities and condemnations at slaughter.

Climate change is anticipated to have the greatest, and most immediate (pre-2020s), impact on intensively farmed animals when in transit [12,13,89]. Given that DOA/DIL was the only condition that tended to increase with outdoor temperature (more than 19°C), our findings support such expectations, at least in terms of losses at slaughter/processing. As maximum daily temperature exceeds 24°C, we may expect large (more than sixfold) increases in the DOA/DIL rates seen at milder temperatures (8–18°C); as was observed in July 2006 (see also [83]). Thus, while improvements in transportation appear to have reduced the impacts of recent summer temperatures in GB, further improvements may be required to lessen the impacts of climate change in the future.

Aside from the risk of increased DOA/DIL rates and on-farm mortality [10] due to heat-stress, the broiler industry might see some benefits from a warming climate through lower condemnation rates of ascites and abnormal colour/fever, in particular. Until then, however, our findings imply that changes are required, on-farm and in-transit, to overcome broiler vulnerabilities to our current, colder, climate.

## 5. Conclusion

In conclusion, the prevalence of some of the most common health and welfare conditions in broiler chickens changes with weather in a temperate climate. Each winter, the welfare of a large number of broilers is compromised, seeming due to inadequate thermal provision on-farm and in-transit, which leads to a considerably greater number of condemned carcasses at slaughter and adds to food waste. With regard to the 16 conditions considered herein, climate change impact assessment and adaptation research should focus on broiler mortality during transportation and lairage. However, we also recommend that the industry takes active steps to reduce the welfare impact of current winter conditions in temperate climates.

## Supplementary Material

File 1: Supporting slaughterhouse data, collected from broiler chickens at approved slaughterhouses in Great Britain. (Sheet a) Monthly time-series for 16 health and welfare conditions, spanning January 2003 - December 2013; (Sheet b) County-level data for 16 health and welfare conditions in broilers slaughtered between 01 Jan 2011 and 31 Dec 2013; (Sheet c) Annual, national-level data for 16 health and welfare conditions, spanning 2011-2013 (inclusive). Data source: Food Standards Agency, UK.

## Footnotes

1 Production quantity (tonnes) of Meat (total) in World (total), 2012/Production quantity (tonnes) of Meat (total) in World (total), 1962. Data source: Food and Agriculture Organization of the United Nations, Statistics Division (FAOSTAT), (http://faostat3.fao.org/download/Q/QL/E), accessed on 11 May 2015.

2 Comparison of aquatic mammal, bovine, pig, poultry, mutton and goat, and ‘other’ meat: Food supply quantity (kg/capita/yr) in World (total), 2011. Data source: FAOSTAT (http://faostat3.fao.org/download/FB/CL/E), accessed on 11 May 2015.

3 Producing animals/slaughtered for chicken meat (1000 head) in World (total), 2013. Data source: FAOSTAT (http://faostat3.fao.org/download/Q/QL/E), accessed on 29 July 2015.

4 Comparison of chickens, rabbits and hares, cattle, ducks, sheep, pigs, goats, turkeys, geese and guinea fowl, buffaloes, horses, asses, pigeons and other birds, camels, other camelids, rodents, mules and animals not otherwise specified: Stocks of Live animals in World (total), 2013. Data source: FAOSTAT (http://faostat3.fao.org/download/Q/QA/E), accessed on 11 December 2015.

5 Unpublished data analyses by first author (following the procedures detailed in §2.2.3) on national monthly slaughterhouse data for Canada, spanning January 1999–May 2014. Data source: Agriculture and Agri-Food Canada (AAFC), 050P Poultry Condemnation Report by Species for Federally Inspected Plants: Chickens (http://aimis-simia.agr.gc.ca/rp/index-eng.cfm?menupos=1.01.04&action=pR&pdctc=&r=133&LANG=EN), accessed on 29 July 2014.

6 National data for 2013.

7 Based on 2010 data.

## Data accessibility

The slaughterhouse datasets supporting this article have been uploaded as the electronic supplementary material. The monthly weather data (© Crown copyright, Met Office) used herein are publically accessible from: http://www.metoffice.gov.uk/public/weather/climate-historic/#?tab=climateHistoric.
